# Subtherapeutic dose lithium treatment induces behavioral and neuropathological changes in 3xTg‐AD Alzheimer's mice

**DOI:** 10.1002/alz70855_107377

**Published:** 2025-12-24

**Authors:** Monique Patricio Singulani, Rosana Camarini, Leda Leme Talib, Luiz Roberto Britto, Orestes Vicente Forlenza

**Affiliations:** ^1^ Centro de Neurociências Translacionais (CNT), Faculdade de Medicina da Universidade de São Paulo, São Paulo, São Paulo, Brazil; ^2^ Laboratory of Neurosciences (LIM27), Departamento e Instituto de Psiquiatria, Hospital das Clínicas, Faculdade de Medicina da Universidade de São Paulo, São Paulo, São Paulo, Brazil; ^3^ Laboratory of Neurochemistry and Behavioral Pharmacology, Departamento de Farmacologia, Instituto de Ciências Biomédicas, Universidade de São Paulo, São Paulo, São Paulo, Brazil; ^4^ Laboratory of Cellular Neurobiology, Departamento de Fisiologia e Biofísica, Instituto de Ciências Biomédicas, Universidade de São Paulo, São Paulo, São Paulo, Brazil

## Abstract

**Background:**

Alzheimer's disease (AD) is the most common cause of dementia and a global health challenge. The efficacy of lithium salts in stabilizing moods and neuroprotective effects has stimulated research into their use in neurodegenerative diseases.

**Method:**

We tested lithium carbonate treatment (1.0g Li_2_CO_3_/kg of chow; 12‐weeks) in 3xTg‐AD and age‐matched Wild‐Type male mice of 12‐month‐old. (CEUA/PROCESS: 1605/2020; 4127240122). Two behavioral test batteries were performed: (1) open‐field test (OFT), novel‐object recognition test (NORT), elevated zero‐maze test (EZMT), and Rotarod test; (2) tail‐suspension test (TST), forced‐swim test (FST), and sucrose preference test (SPT). Immunohistochemistry method was performed to analyze amyloid‐β (Aβ) accumulation (6E10), phosphorylated tau (*p*‐TaupSer199/202), and neuronal viability (neuronal nuclei; NeuN) in areas of the brain. Statistical analysis: two‐way ANOVA followed by Bonferroni post‐hoc test and unpaired t‐test; statistically significant *p* <0.05.

**Result:**

Neurobiological benefits of lithium treatment (mean serum lithium levels; Wild Type+Lithium, 0.22 mmol/L; 3xTg‐AD+Lithium, 0.27 mmol/L) caused improvement/preservation of object recognition memory (object recognition index in short‐ [F_(1, 28)_=8.37; *p* <0.01] and long‐term memory [F_(1, 28)_=7.99; *p* <0.01]). Lithium also caused improvements in anxiety (OFT [F_(1, 28)_=37.15; *p* <0.0001]; EZMT [F_(1, 28)_=19,44; *p* <0,001]), depression (TST [F_(1, 28)_=11.70; *p* <0.01]; FST [F_(1, 28)_=7.96; *p* <0.01]), and anhedonia (SPT [F_(1, 24)_=17,86; *p* <0,001]). The action of lithium was demonstrated in the 3xTgAD+Lithium group when compared to the 3xTg‐AD in reduction in amyloid load (parietal cortex [F(1, 21)=571,80; *p* <0,0001]; hippocampal area CA1 [F_(1, 20)_=417,90; *p* <0,0001]; dentate gyrus [F_(1, 20)_=417,74; *p* <0,001]; subiculum [F_(1, 20)_=1073,00; *p* <0,0001]; and amygdala [F_(1, 22)_=420,00; *p* <0,0001]) and phosphorylated tau (parietal cortex [F_(1, 27)_=845,20; *p* <0,0001]; hippocampal area CA1 [F_(1, 27)_=117,7; *p* <0,0001]; dentate gyrus [F_(1, 27)_=64,89; *p* <0,0001]; subiculum [F_(1, 27)_=76,46; *p* <0,0001]; and amygdala [F_(1, 27)_=; *p* <0,0001]). We also showed that lithium treatment modulated the survival and/or maintenance of neurons (immunostaining for NeuN in parietal cortex [F_(1, 28)_=8,61; *p* <0,01]; hippocampal area CA1 [F_(1, 27)_=70,54; *p* <0,0001]; dentate gyrus [F_(1, 27)_=75,35; *p* <0,0001]; subiculum [F_(1, 26)_=38,67; *p* <0,0001]; and amygdala [F_(1, 27)_=72,77; *p* <0,0001]).

**Conclusion:**

The chronic lithium treatment significantly attenuates the pathological progression of AD, suggesting it as a potential therapeutic drug target for modifying AD. The study was funding by: São Paulo Research Foundation (FAPESP; 2021/06378‐1).

